# 2-Meth­oxy-4-(prop-2-en-1-yl)phenyl benzoate

**DOI:** 10.1107/S1600536813015791

**Published:** 2013-06-12

**Authors:** Mallikarjuna Rao Pichika, Yew Beng Kang, Seik Weng Ng

**Affiliations:** a Department of Pharmaceutical Chemistry, International Medical University, 126 Jalan Bukit Jalil, 57000 Kuala Lumpur, Malaysia; b Department of Chemistry, University of Malaya, 50603 Kuala Lumpur, Malaysia; c Chemistry Department, Faculty of Science, King Abdulaziz University, PO Box 80203 Jeddah, Saudi Arabia

## Abstract

In the title compound, C_17_H_16_O_3_, the benzene rings are twisted by 63.54 (5)°. The twist is similar to that found in the unsubstituted compound, phenyl benzoate. The crystal packing features C—H⋯O hydrogen bonds.

## Related literature

For the structure of phenyl benzoate, see: Shibakami & Sekiya (1995 ▶).
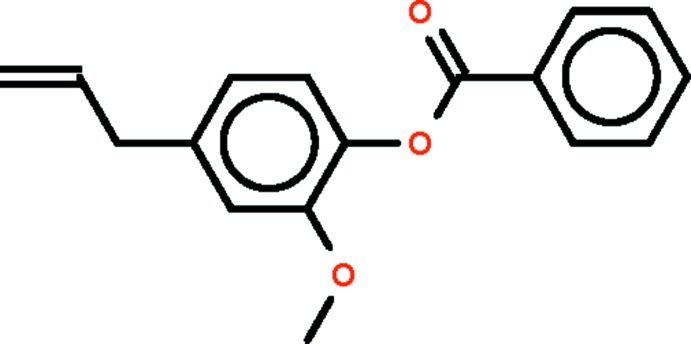


## Experimental

### 

#### Crystal data

C_17_H_16_O_3_
*M* *_r_* = 268.30
Monoclinic, 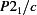

*a* = 9.9334 (6) Å
*b* = 9.5124 (5) Å
*c* = 14.9463 (9) Å
β = 103.405 (6)°
*V* = 1373.81 (14) Å^3^
*Z* = 4
Mo *K*α radiation
μ = 0.09 mm^−1^
*T* = 100 K
0.40 × 0.30 × 0.20 mm

#### Data collection

Agilent SuperNova Dual diffractometer with an Atlas detector
Absorption correction: multi-scan (*CrysAlis PRO*; Agilent, 2013 ▶) *T*_min_ = 0.966, *T*_max_ = 0.983
7024 measured reflections
3168 independent reflections
2242 reflections with *I* > 2σ(*I*)
*R*_int_ = 0.041

#### Refinement

*R*[*F* ^2^ > 2σ(*F* ^2^)] = 0.051
*wR*(*F* ^2^) = 0.138
*S* = 1.05
3168 reflections
181 parameters
H-atom parameters constrained
Δρ_max_ = 0.23 e Å^−3^
Δρ_min_ = −0.29 e Å^−3^

### 

Data collection: *CrysAlis PRO* (Agilent, 2013 ▶); cell refinement: *CrysAlis PRO*; data reduction: *CrysAlis PRO*; program(s) used to solve structure: *SHELXS97* (Sheldrick, 2008 ▶); program(s) used to refine structure: *SHELXL97* (Sheldrick, 2008 ▶); molecular graphics: *X-SEED* (Barbour, 2001 ▶); software used to prepare material for publication: *publCIF* (Westrip, 2010 ▶).

## Supplementary Material

Crystal structure: contains datablock(s) global, I. DOI: 10.1107/S1600536813015791/bt6914sup1.cif

Structure factors: contains datablock(s) I. DOI: 10.1107/S1600536813015791/bt6914Isup2.hkl

Supplementary material file. DOI: 10.1107/S1600536813015791/bt6914Isup3.cml

Additional supplementary materials:  crystallographic information; 3D view; checkCIF report

## Figures and Tables

**Table 1:** Hydrogen-bond geometry (Å, °)

*D*—H⋯*A*	*D*—H	H⋯*A*	*D*⋯*A*	*D*—H⋯*A*
C2—H2⋯O3^i^	0.95	2.55	3.256 (2)	131
C15—H15⋯O3^ii^	0.95	2.54	3.209 (2)	128

Symmetry codes: (i) 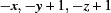; (ii) 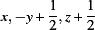.

**Fig. 1.**
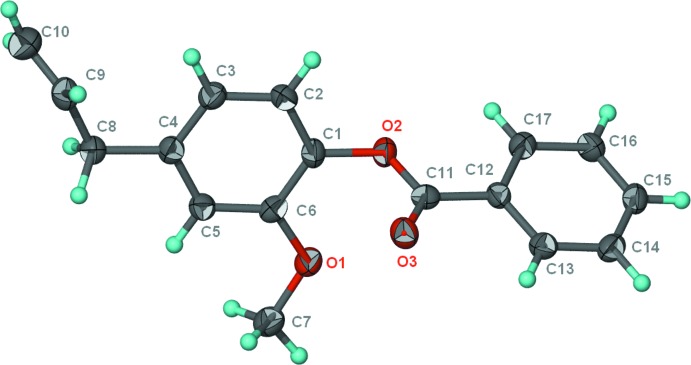
Thermal ellipsoid plot (Barbour, 2001) of C17H16O3 at the 70% probability level; hydrogen atoms are drawn as spheres of arbitrary radius.

## supplementary crystallographic information

### Comment

The title phenyl benzoate (Scheme I, Fig. 1), which possesses an allyl and a methoxy substituent, was synthesized for an evaluation of its pharmaceutical properties as it is an ester derivative of eugenol. The two benzene rings are approximately perpendicular [dihedral angle 63.54 (5)°]. The twist is similar to that found in the unsubstituted compound, phenyl benzoate (Shibakami & Sekiya, 1995).

### Experimental

4-Allyl-2-methoxyphenol (1 mmol), benzoic acid (1 mmol), diethylazodicarboxylate (2 mmol) and triphenylphosphine (2 mmol) were heated in THF (10 ml) for 2 h. The solid material extracted with dichloromethane. The dichloromethane solution was eluted through a silica gel column by using an *n*-hexane–ethyl acetate (95: 5 *v*/*v*) solvent system. Slow evaporation of the solution yielded large colorless crystals.

### Refinement

H-atoms were placed in calculated positions [C–H 0.95 to 0.98 Å, *U*_iso_(H) 1.2 to 1.5*U*_eq_(C)] and were included in the refinement in the riding model approximation.

### Crystal data

**Table:**

C_17_H_16_O_3_	*F*(000) = 568
*M**_r_* = 268.30	*D*_x_ = 1.297 Mg m^−^^3^
Monoclinic, *P*2_1_/*c*	Mo *K*α radiation, λ = 0.71073 Å
Hall symbol: -P 2ybc	Cell parameters from 1957 reflections
*a* = 9.9334 (6) Å	θ = 3.0–27.5°
*b* = 9.5124 (5) Å	µ = 0.09 mm^−^^1^
*c* = 14.9463 (9) Å	*T* = 100 K
β = 103.405 (6)°	Prism, colorless
*V* = 1373.81 (14) Å^3^	0.40 × 0.30 × 0.20 mm
*Z* = 4	

### Data collection

**Table:**

Agilent SuperNova Dual diffractometer with an Atlas detector	3168 independent reflections
Radiation source: SuperNova (Mo) X-ray Source	2242 reflections with *I* > 2σ(*I*)
Mirror monochromator	*R*_int_ = 0.041
Detector resolution: 10.4041 pixels mm^-1^	θ_max_ = 27.6°, θ_min_ = 3.0°
ω scan	*h* = −10→12
Absorption correction: multi-scan (*CrysAlis PRO*; Agilent, 2013)	*k* = −9→12
*T*_min_ = 0.966, *T*_max_ = 0.983	*l* = −19→16
7024 measured reflections	

### Refinement

**Table:**

Refinement on *F*^2^	Primary atom site location: structure-invariant direct methods
Least-squares matrix: full	Secondary atom site location: difference Fourier map
*R*[*F*^2^ > 2σ(*F*^2^)] = 0.051	Hydrogen site location: inferred from neighbouring sites
*wR*(*F*^2^) = 0.138	H-atom parameters constrained
*S* = 1.05	*w* = 1/[σ^2^(*F*_o_^2^) + (0.0572*P*)^2^ + 0.1625*P*] where *P* = (*F*_o_^2^ + 2*F*_c_^2^)/3
3168 reflections	(Δ/σ)_max_ = 0.001
181 parameters	Δρ_max_ = 0.23 e Å^−^^3^
0 restraints	Δρ_min_ = −0.29 e Å^−^^3^

### Fractional atomic coordinates and isotropic or equivalent isotropic displacement parameters (Å^2^)

**Table:**

	*x*	*y*	*z*	*U*_iso_*/*U*_eq_	
O1	0.40190 (12)	0.28310 (12)	0.60263 (8)	0.0266 (3)	
O2	0.22747 (12)	0.48286 (12)	0.63406 (8)	0.0241 (3)	
O3	0.09998 (13)	0.28409 (12)	0.60909 (8)	0.0266 (3)	
C1	0.25534 (17)	0.47175 (17)	0.54614 (11)	0.0216 (4)	
C2	0.19517 (17)	0.56488 (17)	0.47895 (12)	0.0232 (4)	
H2	0.1302	0.6319	0.4904	0.028*	
C3	0.22950 (17)	0.56121 (18)	0.39346 (12)	0.0236 (4)	
H3	0.1877	0.6257	0.3467	0.028*	
C4	0.32409 (17)	0.46399 (17)	0.37666 (11)	0.0216 (4)	
C5	0.38323 (17)	0.36863 (17)	0.44573 (11)	0.0220 (4)	
H5	0.4472	0.3007	0.4341	0.026*	
C6	0.35012 (17)	0.37154 (17)	0.53098 (11)	0.0218 (4)	
C7	0.48978 (19)	0.1729 (2)	0.58544 (13)	0.0305 (4)	
H7A	0.5215	0.1171	0.6415	0.046*	
H7B	0.5699	0.2133	0.5669	0.046*	
H7C	0.4384	0.1124	0.5361	0.046*	
C8	0.36682 (18)	0.45729 (18)	0.28547 (11)	0.0241 (4)	
H8A	0.4647	0.4870	0.2957	0.029*	
H8B	0.3613	0.3583	0.2645	0.029*	
C9	0.2825 (2)	0.5455 (2)	0.21071 (12)	0.0300 (4)	
H9	0.1877	0.5214	0.1888	0.036*	
C10	0.3292 (3)	0.6537 (2)	0.17302 (14)	0.0429 (5)	
H10A	0.4234	0.6809	0.1931	0.052*	
H10B	0.2690	0.7049	0.1255	0.052*	
C11	0.15078 (17)	0.37820 (17)	0.65969 (12)	0.0215 (4)	
C12	0.13802 (17)	0.39478 (17)	0.75630 (11)	0.0206 (4)	
C13	0.08089 (17)	0.28421 (18)	0.79540 (12)	0.0233 (4)	
H13	0.0493	0.2026	0.7601	0.028*	
C14	0.06989 (18)	0.29291 (19)	0.88614 (12)	0.0264 (4)	
H14	0.0324	0.2164	0.9133	0.032*	
C15	0.11346 (18)	0.41292 (18)	0.93709 (12)	0.0261 (4)	
H15	0.1064	0.4185	0.9993	0.031*	
C16	0.16721 (18)	0.52473 (18)	0.89742 (12)	0.0262 (4)	
H16	0.1947	0.6079	0.9320	0.031*	
C17	0.18109 (18)	0.51586 (18)	0.80734 (12)	0.0233 (4)	
H17	0.2198	0.5920	0.7806	0.028*	

### Atomic displacement parameters (Å^2^)

**Table:**

	*U*^11^	*U*^22^	*U*^33^	*U*^12^	*U*^13^	*U*^23^
O1	0.0297 (7)	0.0284 (7)	0.0225 (6)	0.0055 (6)	0.0076 (5)	0.0056 (5)
O2	0.0305 (7)	0.0244 (6)	0.0196 (6)	−0.0024 (5)	0.0102 (5)	−0.0015 (5)
O3	0.0295 (7)	0.0267 (7)	0.0244 (7)	−0.0031 (6)	0.0082 (5)	−0.0049 (5)
C1	0.0244 (9)	0.0236 (9)	0.0183 (8)	−0.0042 (7)	0.0078 (7)	−0.0027 (7)
C2	0.0261 (9)	0.0196 (8)	0.0255 (9)	0.0013 (7)	0.0089 (7)	−0.0018 (7)
C3	0.0268 (9)	0.0228 (8)	0.0210 (9)	−0.0002 (8)	0.0048 (7)	0.0019 (7)
C4	0.0247 (9)	0.0211 (8)	0.0193 (8)	−0.0039 (7)	0.0054 (7)	−0.0022 (7)
C5	0.0208 (9)	0.0215 (8)	0.0251 (9)	0.0002 (7)	0.0079 (7)	0.0000 (7)
C6	0.0219 (8)	0.0215 (8)	0.0212 (8)	−0.0028 (7)	0.0032 (7)	0.0027 (7)
C7	0.0315 (10)	0.0299 (10)	0.0310 (10)	0.0054 (9)	0.0094 (8)	0.0073 (8)
C8	0.0283 (9)	0.0243 (9)	0.0210 (9)	0.0010 (8)	0.0081 (7)	0.0000 (7)
C9	0.0360 (10)	0.0338 (10)	0.0212 (9)	0.0042 (9)	0.0087 (8)	−0.0002 (8)
C10	0.0651 (15)	0.0344 (11)	0.0321 (11)	0.0090 (11)	0.0171 (11)	0.0076 (9)
C11	0.0210 (8)	0.0197 (8)	0.0232 (9)	0.0028 (7)	0.0039 (7)	0.0016 (7)
C12	0.0214 (8)	0.0223 (8)	0.0189 (8)	0.0045 (7)	0.0060 (6)	0.0010 (7)
C13	0.0230 (9)	0.0218 (9)	0.0255 (9)	0.0016 (7)	0.0066 (7)	0.0010 (7)
C14	0.0263 (9)	0.0260 (9)	0.0291 (10)	0.0026 (8)	0.0109 (7)	0.0070 (8)
C15	0.0285 (9)	0.0315 (10)	0.0199 (9)	0.0070 (8)	0.0087 (7)	0.0033 (8)
C16	0.0319 (10)	0.0243 (9)	0.0232 (9)	0.0011 (8)	0.0079 (8)	−0.0030 (7)
C17	0.0254 (9)	0.0221 (9)	0.0230 (9)	−0.0012 (7)	0.0068 (7)	0.0008 (7)

### Geometric parameters (Å, º)

**Table:**

O1—C6	1.3652 (19)	C8—H8A	0.9900
O1—C7	1.425 (2)	C8—H8B	0.9900
O2—C11	1.361 (2)	C9—C10	1.309 (3)
O2—C1	1.4084 (19)	C9—H9	0.9500
O3—C11	1.205 (2)	C10—H10A	0.9500
C1—C2	1.368 (2)	C10—H10B	0.9500
C1—C6	1.395 (2)	C11—C12	1.487 (2)
C2—C3	1.397 (2)	C12—C13	1.387 (2)
C2—H2	0.9500	C12—C17	1.393 (2)
C3—C4	1.382 (2)	C13—C14	1.388 (2)
C3—H3	0.9500	C13—H13	0.9500
C4—C5	1.397 (2)	C14—C15	1.385 (2)
C4—C8	1.520 (2)	C14—H14	0.9500
C5—C6	1.388 (2)	C15—C16	1.384 (2)
C5—H5	0.9500	C15—H15	0.9500
C7—H7A	0.9800	C16—C17	1.387 (2)
C7—H7B	0.9800	C16—H16	0.9500
C7—H7C	0.9800	C17—H17	0.9500
C8—C9	1.490 (2)		
			
C6—O1—C7	116.55 (13)	C4—C8—H8B	108.5
C11—O2—C1	116.84 (13)	H8A—C8—H8B	107.5
C2—C1—C6	121.32 (15)	C10—C9—C8	124.85 (19)
C2—C1—O2	119.29 (15)	C10—C9—H9	117.6
C6—C1—O2	119.27 (14)	C8—C9—H9	117.6
C1—C2—C3	119.82 (16)	C9—C10—H10A	120.0
C1—C2—H2	120.1	C9—C10—H10B	120.0
C3—C2—H2	120.1	H10A—C10—H10B	120.0
C4—C3—C2	120.21 (16)	O3—C11—O2	123.18 (15)
C4—C3—H3	119.9	O3—C11—C12	124.81 (16)
C2—C3—H3	119.9	O2—C11—C12	112.02 (14)
C3—C4—C5	119.15 (15)	C13—C12—C17	120.00 (15)
C3—C4—C8	122.27 (15)	C13—C12—C11	117.65 (15)
C5—C4—C8	118.58 (15)	C17—C12—C11	122.34 (15)
C6—C5—C4	121.12 (16)	C12—C13—C14	119.94 (16)
C6—C5—H5	119.4	C12—C13—H13	120.0
C4—C5—H5	119.4	C14—C13—H13	120.0
O1—C6—C5	125.55 (15)	C15—C14—C13	120.07 (16)
O1—C6—C1	116.07 (14)	C15—C14—H14	120.0
C5—C6—C1	118.37 (15)	C13—C14—H14	120.0
O1—C7—H7A	109.5	C16—C15—C14	120.03 (16)
O1—C7—H7B	109.5	C16—C15—H15	120.0
H7A—C7—H7B	109.5	C14—C15—H15	120.0
O1—C7—H7C	109.5	C15—C16—C17	120.27 (16)
H7A—C7—H7C	109.5	C15—C16—H16	119.9
H7B—C7—H7C	109.5	C17—C16—H16	119.9
C9—C8—C4	115.06 (14)	C16—C17—C12	119.65 (16)
C9—C8—H8A	108.5	C16—C17—H17	120.2
C4—C8—H8A	108.5	C12—C17—H17	120.2
C9—C8—H8B	108.5		
			
C11—O2—C1—C2	111.20 (17)	C3—C4—C8—C9	−10.0 (2)
C11—O2—C1—C6	−72.7 (2)	C5—C4—C8—C9	170.21 (15)
C6—C1—C2—C3	−0.5 (3)	C4—C8—C9—C10	114.8 (2)
O2—C1—C2—C3	175.52 (14)	C1—O2—C11—O3	−4.8 (2)
C1—C2—C3—C4	−0.1 (3)	C1—O2—C11—C12	175.22 (13)
C2—C3—C4—C5	0.8 (2)	O3—C11—C12—C13	9.7 (2)
C2—C3—C4—C8	−178.92 (15)	O2—C11—C12—C13	−170.29 (14)
C3—C4—C5—C6	−1.0 (2)	O3—C11—C12—C17	−170.28 (16)
C8—C4—C5—C6	178.77 (15)	O2—C11—C12—C17	9.7 (2)
C7—O1—C6—C5	−4.6 (2)	C17—C12—C13—C14	−1.6 (3)
C7—O1—C6—C1	174.89 (15)	C11—C12—C13—C14	178.36 (15)
C4—C5—C6—O1	179.88 (15)	C12—C13—C14—C15	1.2 (3)
C4—C5—C6—C1	0.4 (2)	C13—C14—C15—C16	0.4 (3)
C2—C1—C6—O1	−179.15 (15)	C14—C15—C16—C17	−1.7 (3)
O2—C1—C6—O1	4.8 (2)	C15—C16—C17—C12	1.3 (3)
C2—C1—C6—C5	0.4 (2)	C13—C12—C17—C16	0.4 (3)
O2—C1—C6—C5	−175.68 (14)	C11—C12—C17—C16	−179.62 (15)

### Hydrogen-bond geometry (Å, º)

**Table:**

*D*—H···*A*	*D*—H	H···*A*	*D*···*A*	*D*—H···*A*
C2—H2···O3^i^	0.95	2.55	3.256 (2)	131
C15—H15···O3^ii^	0.95	2.54	3.209 (2)	128

Symmetry codes: (i) −*x*, −*y*+1, −*z*+1; (ii) *x*, −*y*+1/2, *z*+1/2.
